# Synthesis and Comparative Investigation of *Ortho*-, *Meta*-, and *Para*-Carboxyphenylmaleimide–Styrene Copolymers

**DOI:** 10.3390/polym18121507

**Published:** 2026-06-16

**Authors:** Shahana Guliyeva, Aygun Alikhanova, Eldar Garaev, Jamila Yusifova, Gaëtan Herbette, Maxime Florent, Bakhtiyar Mammadov

**Affiliations:** 1 Department of Pharmaceutical Toxicology and Chemistry, Faculty of Pharmacy, Azerbaijan Medical University, Baku AZ1022, Azerbaijan; eldarqar@gmail.com (E.G.); camilya@inbox.ru (J.Y.); 2 Institute of Polymer Materials, Ministry of Science and Education of the Republic of Azerbaijan, Sumgait AZ5004, Azerbaijan; aygun81@mail.ru (A.A.); ipoma@science.az (B.M.); 3 Institut de Chimie des Substances Naturelles, CNRS, 91198 Gif-sur-Yvette, France; gaetan.herbette@univ-amu.fr; 4 French-Azerbaijani University (UFAZ), Baku AZ1000, Azerbaijan

**Keywords:** *o*-, *m*-, *p*-carboxyphenylmaleinimides, styrene, synthesis, copolymerization, antibacterial and antifungal activity

## Abstract

The copolymerization of biologically active N-(carboxyphenyl)maleimides with styrene was systematically investigated to elucidate the effect of positional isomerism (*ortho*-, *meta*-, and *para*-) on monomer reactivity and copolymer properties. Reactivity ratios (r_1_, r_2_) were determined using the Fineman–Ross method, and Q–e parameters were evaluated within the Alfrey–Price framework, revealing distinct electronic effects governing copolymerization behavior. Increasing the maleimide fraction in the feed resulted in decreased copolymer yield, intrinsic viscosity, molecular weight, and glass transition temperature, while all copolymers remained styrene-rich, indicating preferential styrene propagation. Comprehensive structural characterization (NMR, FTIR, and UV–Vis) confirmed successful incorporation of both monomer units. Rheological analysis demonstrated a clear viscosity trend (*ortho* > *meta* > *para*), highlighting the influence of substituent position on chain interactions and macromolecular architecture. Thermal analysis (TGA/DTA) showed good thermal stability up to 250–300 °C. Notably, the copolymers exhibited significant antibacterial and antifungal activity, with maximum inhibition observed against *Candida albicans*. This study establishes a direct correlation between substituent position and structure–property relationships, providing new insights for the rational design of functional styrenic copolymers with potential applications in antimicrobial and biomedical materials.

## 1. Introduction

Styrene-based copolymers modified with functional comonomers remain a central topic in polymer chemistry due to their tunable physical properties and broad industrial significance. In particular, maleimide-containing copolymers have been extensively studied as high-performance materials exhibiting enhanced thermal stability, mechanical strength, and chemical resistance compared to conventional polystyrene [1,2,3,4,5]. Functional maleimide monomers are of considerable interest in modern polymer chemistry due to their combination of high reactivity and the ability to introduce various functional groups into the macromolecular chain. The introduction of a carboxyl group into the phenyl ring of N-phenylmaleimides is an effective structural factor that increases the polarity of the monomers, enhances intermolecular interactions via hydrogen bonding, and improves the compatibility of copolymers with thermoplastics. These effects lead to improved adhesion properties and increased thermal stability of polymeric materials [6,7,8,9,10,11,12].

The incorporation of aromatic maleimide derivatives imparts additional rigidity to the polymer backbone, while functional substituents enable further modification through intermolecular interactions or post-polymerization reactions. Among these derivatives, carboxyl-functionalized maleimides are of particular interest, as the presence of the carboxyl group can influence copolymer composition, chain mobility, and thermal behavior [13,14,15,16,17,18,19].

In our previous studies, the synthesis of copolymers containing biologically active groups based on maleic acid bisimides was reported, along with their application as bioactive polymer additives in the production of antibacterial composite materials [20,21,22,23,24].

Although styrene–maleimide systems have been widely reported in the literature, the influence of positional isomerism of functional groups on the aromatic ring has not been sufficiently investigated. The position of the carboxyl group (two-, three-, and four-) can significantly affect monomer reactivity and copolymer structure; however, systematic comparative studies are largely lacking, which underscores the relevance of the present work. Steric and electronic effects associated with *ortho*-, *meta*-, and *para*-substitution may markedly alter monomer reactivity and the microstructure of the resulting copolymers. Therefore, a systematic comparison of styrene copolymers containing *o*-, *m*-, and *p*-carboxyphenylmaleimide units is necessary to establish structure–property relationships [25].

The copolymerization of maleimides with styrene represents a typical example of the interaction between an electron-deficient dienophile (maleimide) and an electron-donating vinyl monomer (styrene), which generally leads to the formation of alternating copolymers. The introduction of a carboxyl group into the phenyl ring of N-phenylmaleimides results in increased monomer polarity, enhanced intermolecular hydrogen-bonding capability, improved compatibility of the copolymer with engineering thermoplastics, as well as improved adhesion properties and thermal stability.

The aim of this study is to synthesize these isomeric maleimide monomers, prepare their copolymers with styrene under identical conditions, and evaluate the effect of substituent position on structural and thermal properties.

## 2. Materials and Methods

### 2.1. Materials

*Ortho*-, *meta*-, and *para*-carboxyphenylmaleimides were synthesized using a modified method described in the literature via the corresponding anhydrides, followed by cyclization to the imide [26]. Styrene (ST) was first purified to remove the inhibitor by vacuum distillation. Benzeneoyl peroxide (BP, Sigma-Aldrich, St. Louis, MO, USA) was used as the initiator. All solvents were of analytical grade (Merck, Darmstadt, Germany).

### 2.2. Copolymerization

Radical copolymerization of N-(carboxyphenyl)maleimides with ST was carried out in sealed glass ampoules in methyl ethyl ketone solution in the presence of 0.2 wt.% BP at 70 °C for 8 h under an inert atmosphere. The total concentration of the initial monomer mixture was kept constant at 1.0 mol·L^−1^. The concentration of BP was 2 wt% of the total monomer mass. The monomer ratio varies as shown in Table 2. The resulting copolymers were precipitated from the reaction mixture with n-hexane, washed several times with hot benzene and diethyl ether, and dried under vacuum (30–40 mmHg) at 40 °C to constant weight. The copolymers were obtained as yellow powders and were readily soluble in methyl ethyl ketone and chlorinated organic solvents. The yield was 83–85%. The copolymer synthesized from the initial reaction mixture with a 1:2 (mol%) composition has the following properties: melting temperatures *o*-275 °C, *m*-256 °C, and *p*-265 °C.

### 2.3. Methods

The obtained copolymers were characterized by using a variety of techniques. The composition and structure of the copolymers were investigated using Nuclear Magnetic Resonance (NMR) spectroscopy (^1^H, ^13^C, and 2D NMR (COSY, HMBC)), FTIR, and UV spectroscopy [27,28]. All NMR spectra were recorded in DMSO-d6 or CDCl3 (concentration: 10 mg·mL^−1^) using a Bruker Avance III 600 MHz spectrometer equipped with a TXI probe (Bruker BioSpin GmbH, Rheinstetten, Germany).

The IR spectra of the samples were recorded on an Agilent Cary 630 FTIR spectrometer (Agilent Technologies, Santa Clara, CA, USA) using a ZnSe crystal in the wavenumber range of 600–4000 cm^−1^. UV–Vis spectra were recorded in the wavelength range of 200–800 nm using an Agilent Technologies UV–Vis spectrophotometer [29,30].

The molecular weights (MW) and molecular weight distribution (MWD) parameters of the copolymers were determined by exclusion liquid chromatography (ELC) on a high-performance liquid chromatograph manufactured by Kovo (Prague, Czech Republic) equipped with a refractometric detector. For MW determination, two columns (3.3 × 150 mm) connected in series and packed with Separon-SGX sorbent (particle size 7 μm, pore sizes 100 and 200 Å) were used. DMF was employed as the eluent at a flow rate of 0.3 mL·min^−1^ and a temperature of 20–25 °C. The calibration curve (log M vs. V_R) over the molecular weight range of (1.5–100) × 10^3^ was obtained using polystyrene standards. The MWD chromatograms were interpreted according to the method described in [31].

The rheological properties of styrene copolymers containing positional isomers of carboxyphenylmaleimide (CPMI)—*ortho*-, *meta*-, and *para*-—were investigated using an MCR 302 rotational rheometer (Anton Paar GmbH, Graz, Austria) [32,33]. Measurements were performed in steady shear and oscillatory modes at temperatures between 180 and 220 °C.

Thermal analysis was carried out using a STA 449 F3 Jupiter simultaneous thermal analyzer (NETZSCH-Gerätebau GmbH, Selb, Germany) at a heating rate of 20 K·min^−1^ under a nitrogen atmosphere [34,35].

### 2.4. The Study of Antibacterial Properties of Copolymers Based on o-, m-, p-CPhMI–Styrene

The antimicrobial activity of synthesized monomers was evaluated using the disk-diffusion method [36]. The disk-diffusion method was used to study the antibacterial and antifungal properties of the presented copolymers [25,37,38,39]. To evaluate antimicrobial activity, a range of microorganisms was used, including *Staphylococcus aureus* (Gram-positive), *Escherichiacoli* and *Pseudomonas aeruginosa* (Gram-negative), *Bacillus anthracoides* (spore-forming Gram-positive), *Klebsiellapneumoniae* (capsular Gram-negative) and *Candida albicans* (fungus). Microbial suspensions were prepared from fresh daily cultures by diluting approximately 1 mL of microbial cells in 1 mL of sterile physiological solution. Each suspension was evenly spread onto Petri dishes containing appropriate growth media—Meat-Peptone Agar (APA) for bacteria and Sabouraud Agar for fungi. Excess suspension was removed with a pipette and safely discarded in disinfectant. The inoculated plates were allowed to dry at 37 °C for 10 min. Sterile disks, preheated on a sterile surface for 3–5 min, were placed gently onto the inoculated media using sterile forceps, ensuring good contact with the surface. Plates with APA were incubated at 37 °C, while those with Sabouraud medium were incubated at 28 °C. The active compounds diffused into the agar from the disks, inhibiting microbial growth. After 24–48 h of incubation, the plates were examined, and the zones of inhibition were recorded. The following three types of fungi were used in the study: *Aspergillus niger*, *Aspergillus ochraceus*, and *Fusariumnugamai*, grown on a nutrient medium with agar-saburo. The working chamber was pre-disinfected with alcohol, after which a UV-C germicidal lamp (254 nm; Philips, Amsterdam, The Netherlands) was switched on to maintain sterility. The culture medium was melted on a heating surface and poured into Petri dishes under an open flame to minimize contamination. Each dish was filled to approximately one-third of its volume. After the medium had dried, the fungal mycelium was inoculated using sterile lancets, placing the plastic samples under investigation next to the cultures. Incubation was carried out at 28 °C for 1 month to study the effect of fungi on plastic.

Preparation of the medium was as follows: 65.5 g of agar-saburo was dissolved in 1 L of distilled water and autoclaved for 15 min at 1 atm. After autoclaving the medium was cooled to a temperature suitable for filling and filtration.

The activity of *o*-, *m*-, *p*-carboxyphenylmaleinimide–styrene copolymers against selected microorganism cultures (*S. aureus*, *E. coli*, *P. aeruginoza*, *C. albicans*, *K. pneumoniae*, and *B. anthracoides*) was checked and it was determined that its effect on fungal cells (*C. albicans*-microbe-free zone diameter 15–18 mm) is more effective.

## 3. Result and Discussion

**Scheme 1:**
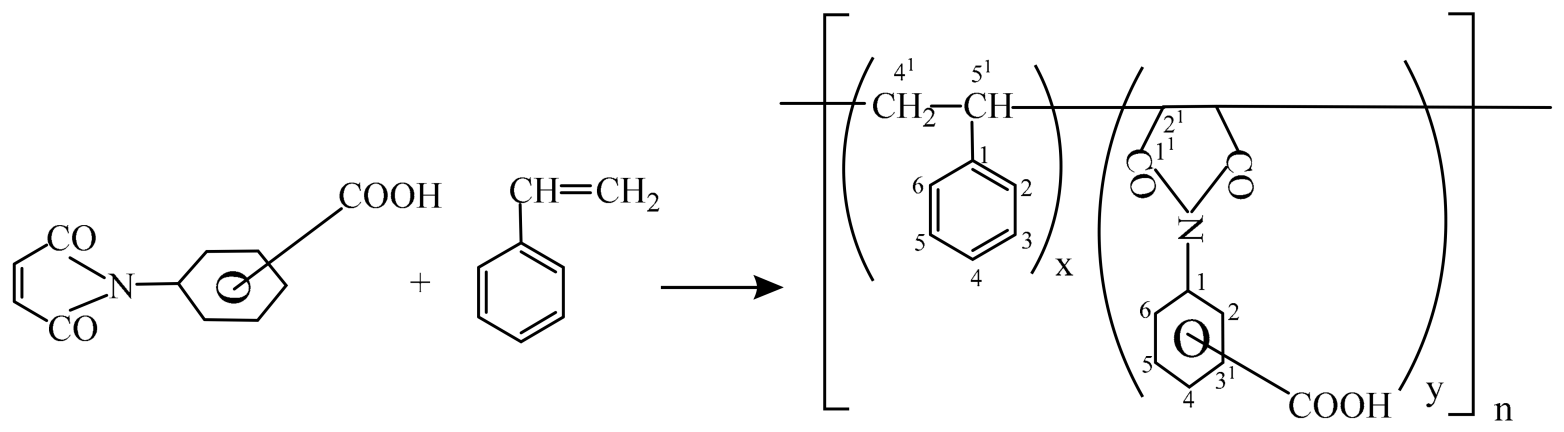
Copolymerization reactions of styrene with *ortho*-, *meta*-, and *para*-carboxyphenylmaleimides.

Copolymerization reactions of styrene (M_1_) with *ortho*-, *meta*-, and *para*-carboxyphenylmaleimide (M_2_) were carried out to obtain a copolymer of styrene and carboxyphenylmaleinimide with antibacterial properties, physico-mechanical and antibacterial properties of the synthesized copolymer samples were investigated according to the methodology specified in [3,6,25]. Solution radical polymerization was chosen because it provides homogeneous reaction conditions, efficient heat transfer, and reproducible copolymer formation, while avoiding the need for complex catalytic systems. The copolymerization reactions of styrene with *ortho*-, *meta*-, and *para*-carboxyphenylmaleimides are shown in Scheme 1.

### 3.1. Composition and Molecular Weight Characteristics

In all cases, the obtained copolymers were enriched in styrene units. An increase in the content of carboxyphenylmaleimides led to a decrease in reaction yield, intrinsic viscosity, and average molecular weight. This effect is attributed to reduced macroradical mobility and increased system polarity.

**Figure 1:**
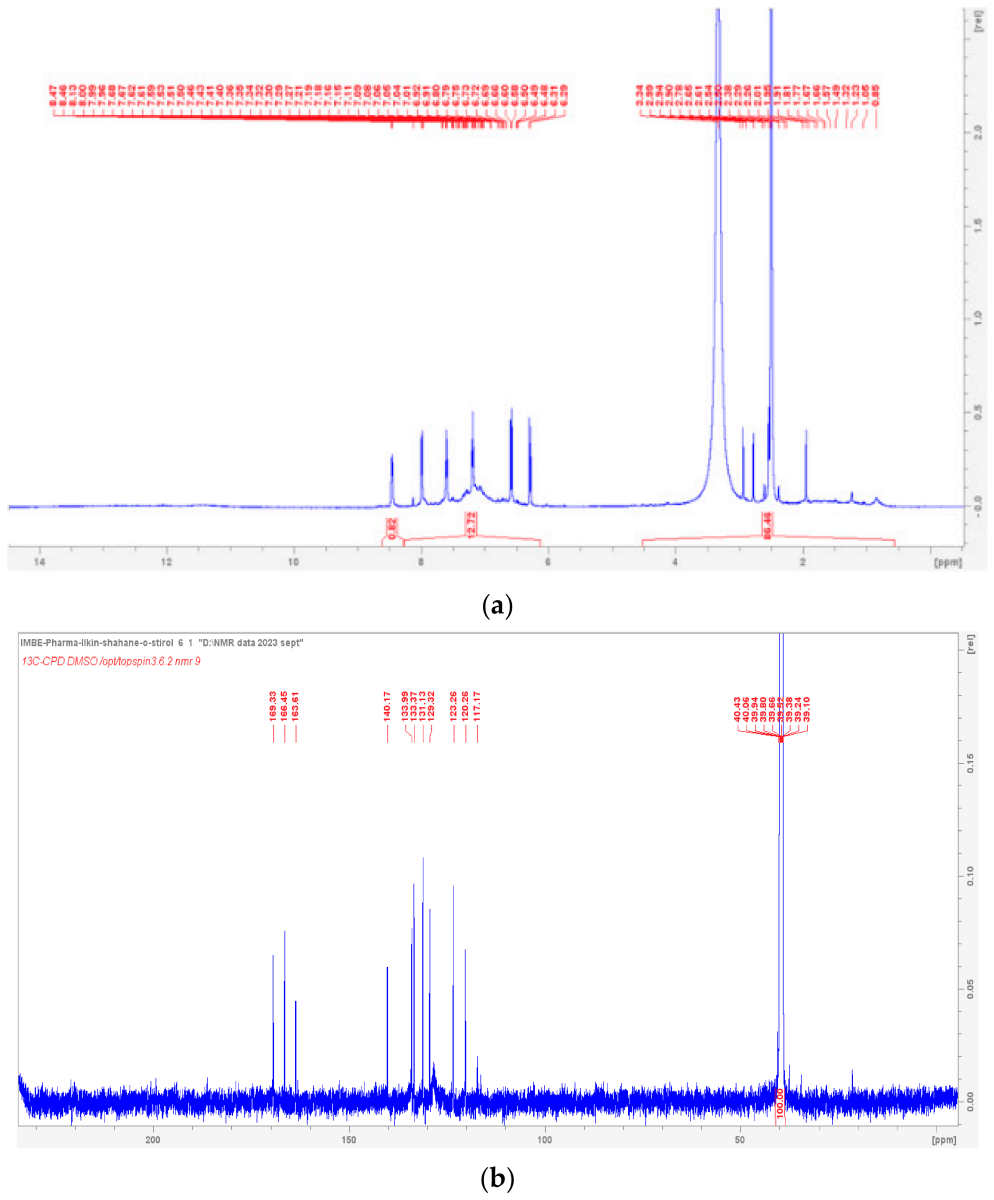
^1^H (**a**), ^13^C (**b**) NMR spectra of *o*-carboxyphenylmaleinimide–styrene copolymer.

**Figure 2:**
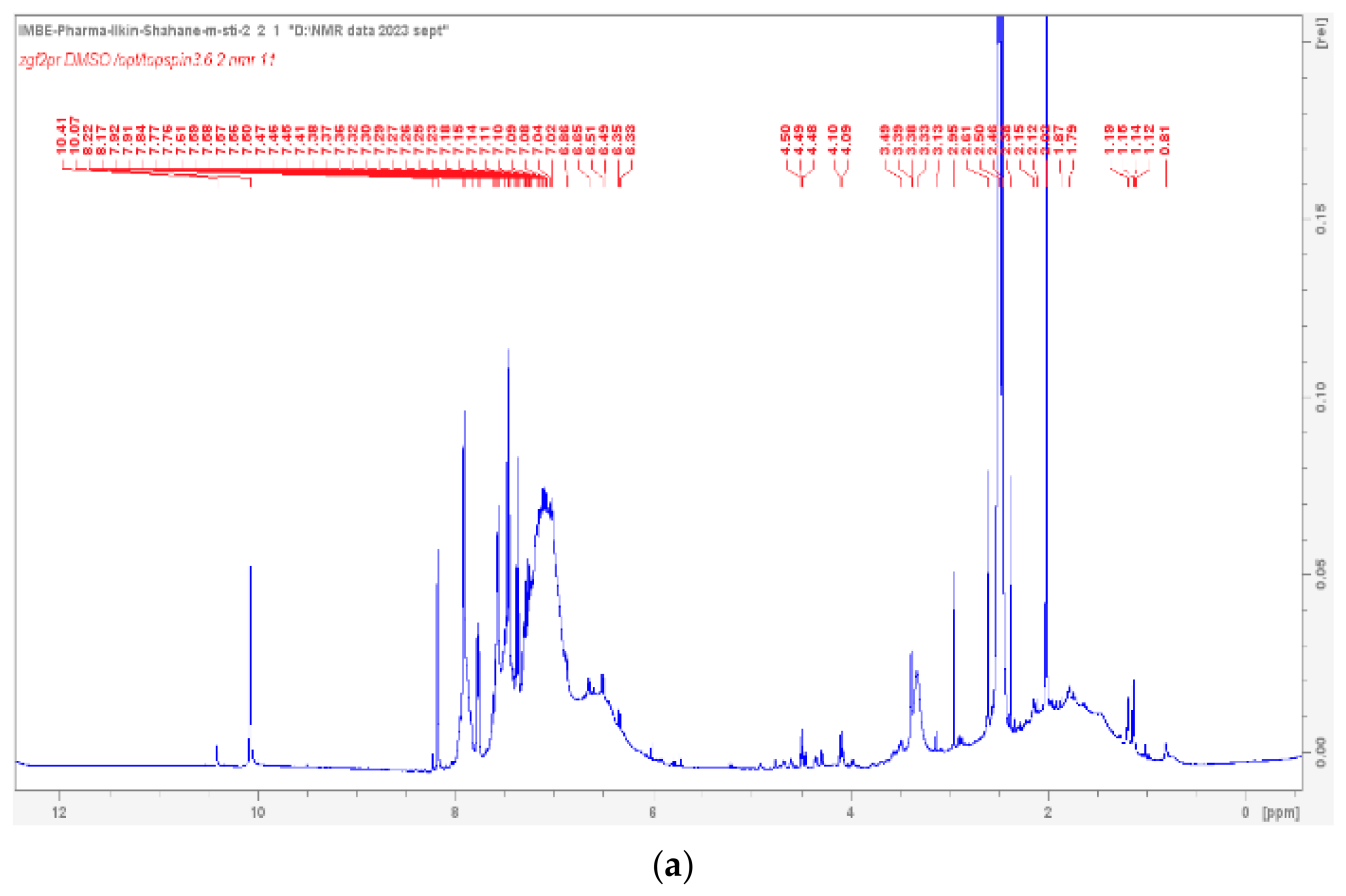

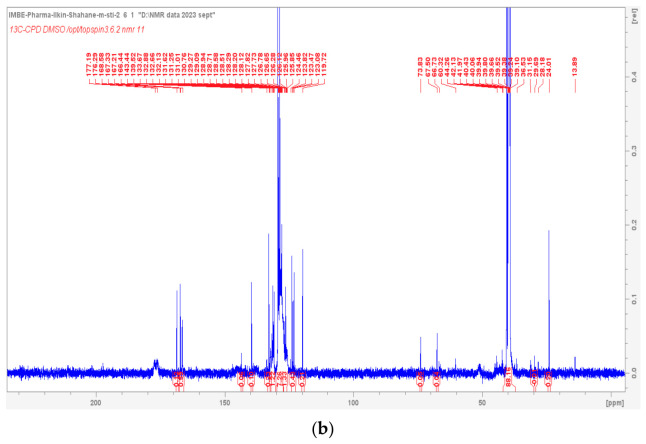
^1^H (**a**), ^13^C (**b**) NMR spectra of *m*-carboxyphenylmaleinimide–styrene copolymer.

**Figure 3:**
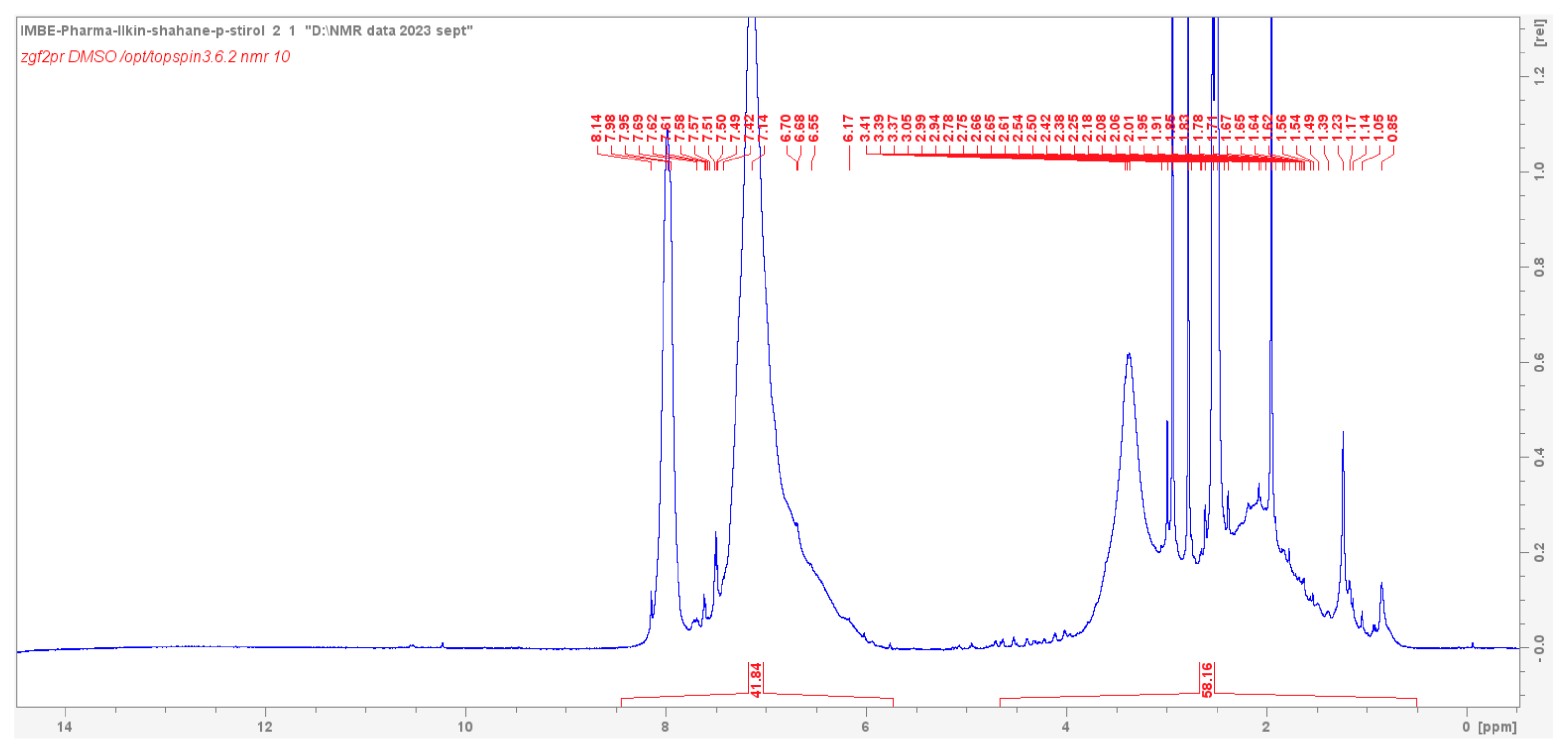
^1^H NMR spectra of *para*-carboxyphenylmaleinimide–styrene copolymer.

The composition and structure of the copolymers were investigated using NMR spectroscopy (^1^H, ^13^C, and 2D NMR (COSY, HMBC)), high-resolution, FTIR and UV spectroscopy. Based on the NMR spectroscopy results, the structures of the *o*-, *m*-, and *p*-carboxyphenylmaleimide–styrene copolymers are shown in Figure 1, Figure 2 and Figure 3.

Figure 1 and Figure 2 present the ^1^H (a) and ^13^C (b) NMR spectra of the *o*-, *m*-CPMI–styrene copolymer. The chemical structure of the copolymer was further confirmed by 2D NMR techniques (COSY and HMBC), which are provided in the Supporting Information.

The ^1^H NMR spectrum recorded in DMSO-d_6_ shows broad aromatic resonances at δ 6.4–8.2 ppm, attributed to phenyl protons of both styrene and *ortho*-carboxyphenylmaleimide units, confirming incorporation of both comonomers. A weak, broad signal at δ ~12.5–13.5 ppm corresponds to the –COOH proton, indicating preservation of the carboxylic acid functionality after polymerization. Broad aliphatic signals at δ 1.2–3.0 ppm are assigned to methine and methylene protons of the saturated polymer backbone. The absence of vinyl proton signals in the δ 5.5–6.8 ppm region confirms complete monomer conversion and successful addition copolymerization.

Overall, the NMR data confirm successful copolymer formation with retention of the –COOH functional group.

The ^13^C NMR of the styrene–*ortho*-carboxyphenylmaleimide copolymer shows carbonyl signals at δC 165–175 ppm (imide and –COOH), aromatic carbons at δC 125–145 ppm, and aliphatic backbone carbons at δC 30–55 ppm. Signal broadening and splitting confirm copolymer formation, incorporation of functional groups, and the polymeric sequence distribution.

Cross-peaks between backbone protons (δH 1.5–3.5 ppm) and aromatic/imide carbons (δC 35–55 ppm) further confirm styrene–maleimide connectivity, supporting a random copolymer architecture. Additionally, broadened and slightly downfield-shifted aromatic correlations are consistent with the *ortho*-substituted carboxyphenyl structure, confirming preservation of the functional maleimide unit within the copolymer.

^1^H and ^13^C NMR chemical shifts for *o*-carboxyphenylmaleimide–styrene copolymers are shown in Table 1.

The *meta*-carboxyphenylmaleimide–styrene copolymer structure was confirmed by ^1^H NMR in DMSO-d_6_. Broad aromatic signals at δ 6.7–7.9 ppm correspond to phenyl protons, while aliphatic peaks at δ 1.2–2.3 ppm arise from the polymer backbone. Signals at δ 3.0–3.7 ppm confirm incorporation of maleimide units. The absence of vinyl proton signals indicates complete polymerization. A weak broad peak at δ ~ 12–13 ppm is assigned to the –COOH proton. The data confirm successful copolymer formation and preservation of functional groups.

^1^H NMR confirms styrene–*para*-carboxyphenylmaleimide copolymer formation. Aromatic (δH 6.5–8.1 ppm; δC 125–145 ppm) and aliphatic (δH 1.2–2.4 ppm; δC 30–45 ppm) signals, along with carbonyl peaks (δC 165–175 ppm), verify complete monomer incorporation and retention of functional groups.

**Figure 4:**
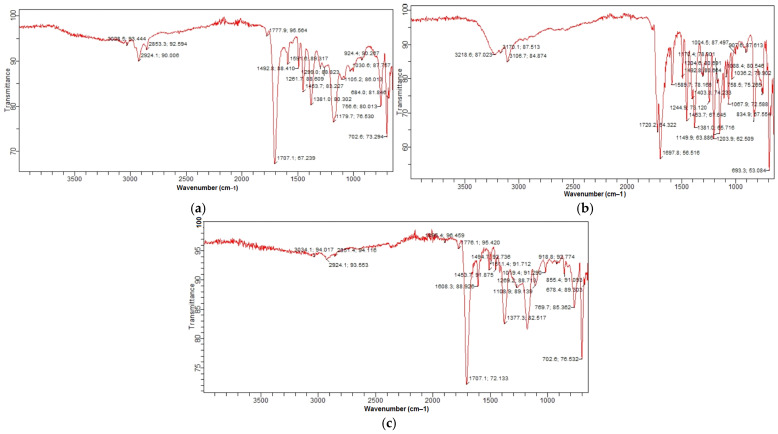
IR spectrum of the *o*- (**a**), *m*- (**b**), *p*-carboxyphenylmaleimides (**c**) –styrene copolymers.

Fourier transform infrared (FTIR) spectroscopy was employed to confirm the chemical structure of the styrene–*meta*-carboxyphenylmaleimide copolymer. Based on the results of IR spectroscopy, the copolymers are shown in Figure 4. The spectrum exhibits characteristic absorptions corresponding to both styrene and maleimide moieties, confirming successful copolymer formation.

A strong absorption band at 1720 cm^−1^ and a shoulder at 1697 cm^−1^ are attributed to the C=O stretching vibrations of the imide carbonyl groups. The band at 1720 cm^−1^ may also include partial contribution from carboxylic acid carbonyl groups. The presence of these two closely spaced carbonyl absorptions is characteristic of the imide functionality and confirms the incorporation of the maleimide unit into the polymer backbone.

A weak and broad absorption feature in the 3200–2500 cm^−1^ region may be associated with the O–H stretching vibration of carboxylic acid groups present in the copolymer structure. The low intensity and poor resolution of this band are attributed to extensive hydrogen bonding and the relatively low concentration of carboxyl functionalities.

Bands appearing in the region of <3000 cm^−1^ correspond to aromatic C–H stretching vibrations, while absorptions at ~2920 and 2850 cm^−1^ are assigned to aliphatic C–H stretching, consistent with the styrene-derived polymer backbone. The aromatic ring stretching vibrations are evident at ~1600–1500 cm^−1^, confirming the presence of phenyl groups from both styrene and phenyl maleimide units.

The absorption observed at ~1370–1385 cm^−1^ is attributed to C–N stretching of the imide ring, further supporting successful maleimide incorporation. Additionally, bands in the region of ~760–700 cm^−1^ correspond to aromatic C–H out-of-plane bending vibrations, consistent with substituted benzene rings.

Notably, the absence of a characteristic alkene C=C stretching band near ~1640 cm^−1^ indicates complete consumption of the vinyl double bonds, confirming successful polymerization.

Overall, the FTIR spectrum provides strong evidence for the formation of a styrene–*o*- (a), *m*- (b), *p*-carboxyphenylmaleimide copolymers containing imide, carboxylic acid, aromatic, and aliphatic functionalities, in agreement with the proposed chemical structure.

**Figure 5:**
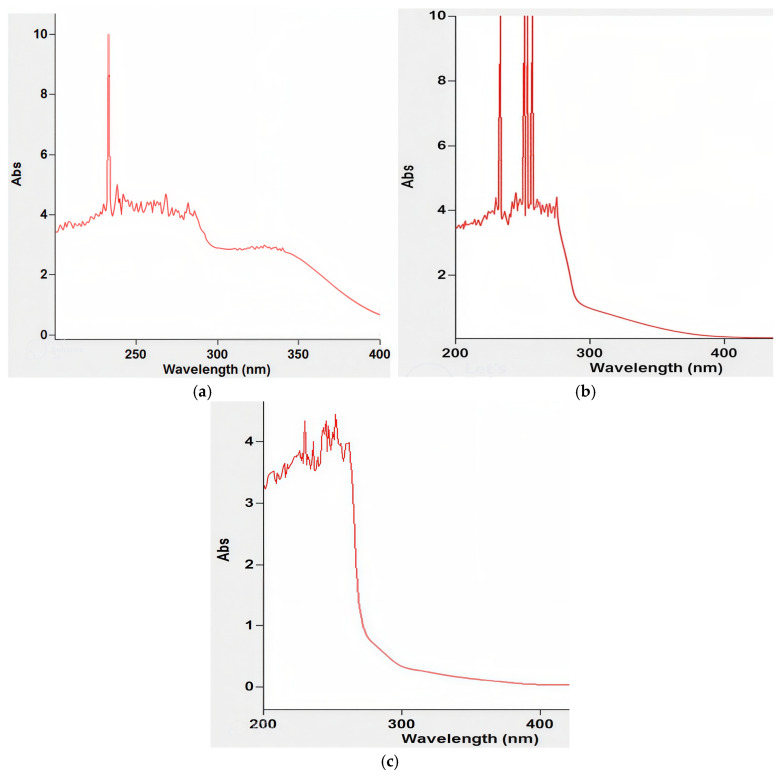
UV spectrum of the *o*- (**a**), *m*- (**b**), *p*-carboxyphenylmaleimides (**c**) –styrene copolymers.

Based on the results of UV spectroscopy, the copolymers are shown in Figure 5.

The *o*-carboxyphenylmaleimide–styrene copolymer shows strong absorption at 240–300 nm from aromatic π → π* transitions and imide C=O contributions. A band at 250–270 nm confirms benzene chromophores, while slight bathochromic shifts indicate interaction between styrene and maleimide units. No absorption above 320–350 nm confirms limited conjugation, verifying successful incorporation of the maleimide and retention of aromatic functionality.

The styrene–/*meta*-carboxyphenylmaleimide copolymer shows intense π → π* absorption at 200–230 nm from aromatic rings and a broad shoulder at 250–290 nm due to aromatic and imide C=O transitions. No absorption above 300 nm indicates the absence of extended conjugation, confirming incorporation of maleimide units and a structurally stable aromatic–imide copolymer.

The styrene–/*para*-carboxyphenylmaleimide copolymer shows strong π → π* absorption at 200–240 nm and a broad 240–290 nm band from aromatic and imide C=O transitions. No absorption above 300 nm indicates localized electronic transitions and absence of extended conjugation, confirming incorporation of aromatic and imide units in a stable copolymer.

### 3.2. Kinetics and Reactivity of Monomers

The radical copolymerization of styrene with *o*-, *m*-, and *p*-carboxyphenylmaleimides was studied to determine the effect of substituent position on reaction kinetics and monomer reactivity. All systems followed pseudo-first-order kinetics and showed a tendency toward alternating copolymerization due to donor–acceptor interactions between styrene and maleimide units. The *para*-isomer exhibited the highest polymerization rate because of lower steric hindrance and better radical stabilization, while the *ortho*-isomer showed the lowest rate due to steric effects and possible intramolecular hydrogen bonding. The meta-isomer displayed intermediate behavior between the two. Overall, both electronic and steric factors significantly influence the copolymerization process and the properties of the resulting materials.

Copolymer composition was evaluated using the Mayo–Lewis copolymerization equation, where *M*_1_ and *M*_2_ represent styrene and carboxyphenylmaleimide monomers, respectively, and *r*_1_ and *r*_2_ are their corresponding reactivity ratios. In this system, styrene behaves as an electron-donating monomer, while carboxyphenylmaleimides act as strong electron-accepting comonomers due to the imide and carboxyl functionalities.

For all positional isomers (*o*-, *m*-, and *p*-CPMI), the reactivity ratio of the maleimide monomer (*r*_2_) is expected to be significantly less than unity (*r*_2_ ≪ 1), reflecting its negligible tendency toward homopropagation. Similarly, the styrene reactivity ratio (*r*_1_) is also less than unity (*r*_1_ < 1), indicating preferential cross-propagation over styrene–styrene addition. This combination of reactivity ratios (*r*_1_·*r*_2_ ≪ 1) is characteristic of strongly alternating copolymerization behavior.

The position of the carboxyphenyl substituent significantly influences reactivity ratios through steric and electronic effects. The *para*-isomer shows the highest tendency toward alternating copolymerization, while the *ortho*-isomer exhibits reduced reactivity due to steric hindrance and intramolecular interactions, with the *meta*-isomer displaying intermediate behavior. At low conversions, the system maintains stable composition, indicating steady-state kinetics and controlled copolymer formation.

Conversion–time analysis shows that the *para*-isomer has the highest copolymerization rate, the *meta*-isomer shows intermediate behavior, and the *ortho*-isomer the slowest. This is due to steric hindrance and possible intramolecular interactions in the *ortho*-isomer, which limit radical addition. In contrast, the *para*-isomer benefits from lower steric effects and better conjugation, enhancing polymerization efficiency.

Copolymers containing different ratios of *o*-, *m*-, *p*-carboxyphenylmaleinimides and elementary acts of styrene were synthesized; simultaneously, the relative activities of the monomers (r_1_ and r_2_) in the copolymerization reactions were determined, and composition–composition and composition–property relationships were studied (Table 1). The obtained and purified polymer sample was separated into fractions by partial dissolution method, and after drying each fraction, elemental analysis was performed. The results of elemental analysis for each fraction were almost the same. This indicates that during copolymerization of *o*-, *m*-, *p*-carboxyphenylmaleinimide with styrene, only one substance was formed, that is, a copolymer of these monomers, i.e., no homopolymer was obtained from the monomers used under the conditions considered.

Values of relative activity of monomers during radical copolymerization of *o*-, *m*-, and *p*-carboxyphenylmaleinimide and styrene shows (Table 2) that styrene has higher relative activity from this pair of monomers. Accounting for the electronic nature of the monomers M_1_ and M_2_, the weak π-complex formed as a result of charge transfer between these monomers during copolymerization plays a crucial role in both the excitation and chain elongation stages. As a result, copolymer macromolecules with a statistical structure consisting of alternating fragments and styrene blocks on the monomer pair are formed.

Table 2 shows the dependence of the copolymer composition on the composition of the initial monomer mixture in the copolymerization reaction of St (M_1_) and o-, *m*-, and *p*-carboxyphenylmaleinimides (M_2_).

**Table 2:** Relative activity of monomers in the copolymerization reactions of St (M_1_) and *o*-, *m*-, *p*-carboxyphenylmaleinimides (M_2_) (solvent–DMF, T = 70 °C, reaction duration −8 h.).

The Composition of the Initial Mixture, mol%		Copolymers	Composition of Copolymers, mol%		Copolymerization Constants			* Q-e Parameters	
M_1_	M_2_		m_1_	m_2_	r_1_	r_2_	r_1_·r_2_	Q_1_	e_1_
90	10	*o*-CPhMI	85.6	14.4					
		*m*-CPhMI	86.2	13.8					
		*p*-CPhMI	86	14					
75	25	*o*-CPhMI	74	26					
		*m*-CPhMI	73	27					
		*p*-CPhMI	73.3	26.7					
50	50	*o*-CPhMI	60	40	0.20	0.10	0.02	0.25	+1.8
		*m*-CPhMI	61	39	0.15	0.08	0.012	0.20	+2.0
		*p*-CPhMI	60.4	39.6	0.10	0.05	0.05	0.22	+1.9
25	75	*o*-CPhMI	52	48					
		*m*-CPhMI	53	47					
		*p*-CPhMI	54	46					
10	90	*o*-CPhMI	50.8	49.2					
		*m*-CPhMI	50.7	49.3					
		*p*-CphMI	51	49					

* Q_1_ and Q_2_ are the reactivity of monomers m_1_ and m_2_ during copolymerization, respectively; e_1_ and e_2_ are values proportional to the excess charges on the radicals derived from these monomers. Q_1_ = 1.0; e_1_ = −0.80.

The copolymerization constants (r_1_ and r_2_) were calculated using the Fineman–Ross equation, and the Q-e parameters were calculated using the formula of Alfrey–Price.

The copolymer composition is enriched in styrene elementary acts in all quantitative values of the primary monomers. This fact can be explained by the low relative activity of carboxyphenylmaleinimide monomer compared to styrene in the radical copolymerization reaction and the high probability of its participation in chain transfer. The effect of their composition on the properties of copolymers obtained in copolymerization reactions of styrene with *o*-, *m*-, and *p*-carboxyphenylmaleinimides is shown in Table 3.

As can be seen from Table 3, as the relative amount of carboxyphenylmaleinimides in the monomer mixture increases, the reaction yield and the characteristic viscosity of copolymers, correspondingly, the average molecular mass and viscosity transition temperature are observed to decrease.

The agreement among experimental conversion–time behavior, steady-state kinetic models, and Mayo–Lewis reactivity considerations confirms that styrene–carboxyphenylmaleimide copolymerization proceeds via a donor–acceptor-driven radical mechanism with isomer-dependent propagation efficiency.

The molecular weights (MWs) and molecular weight distribution (MWD) parameters of the copolymers were determined by size-exclusion chromatography (SEC) using a high-performance liquid chromatograph. The calculations were performed using standard equations:

$${\bar{M}}_{w}=\sum M_{i}\omega_{i};\;\;\;\;{\bar{M}}_{n}=1/(\omega_{i}/\sum M_{i})$$

where *M_i_* is the molecular weight corresponding to the *i*-th fraction of the chromatogram area, and ω*_i_* is the fraction of the area of the i-th segment relative to the total chromatogram area. 

**Figure 6:**
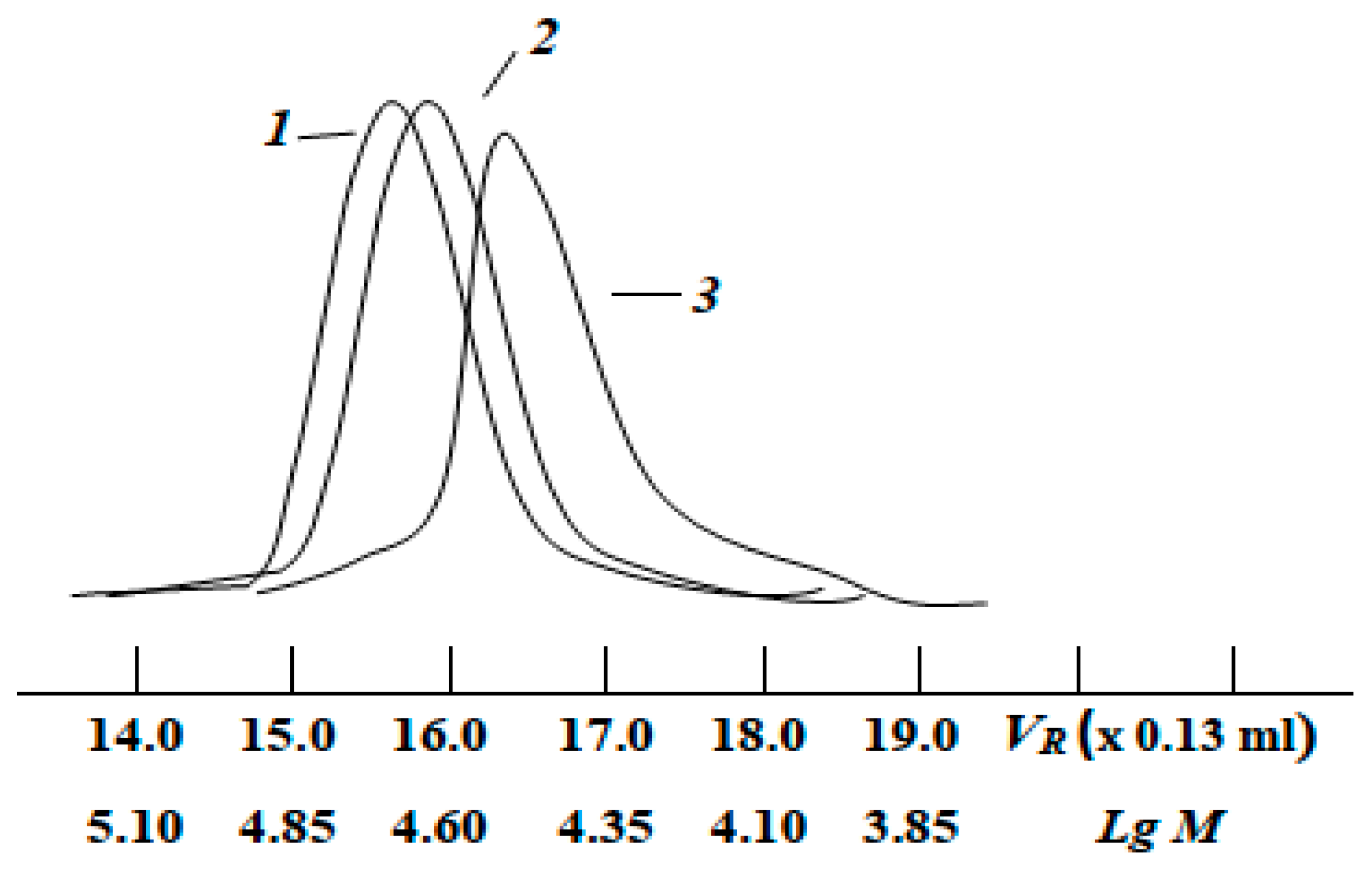
Molecular weight distribution (MWD) curves of styrene copolymers with *p*-, *m*-, and *o*-carboxyphenylmaleimides (curves 1, 2, and 3, respectively). Chromatographic conditions: columns (3.3 × 150 mm) packed with Separon-SGX sorbent (particle size 7 μm; pore sizes 100 and 200 Å); eluent: DMF; flow rate: 0.3 mL·min^−1^; temperature: 20–25 °C; detector: refractive index.

The molecular weight distribution (MWD) of the copolymers was analyzed by gel permeation chromatography (GPC), and the results are shown in Figure 6.

The molecular weight characteristics of the obtained copolymers are summarized in Table 4.

The formation of donor (D)–acceptor (A) type complexes (complexes formed by charge transfer) between St and CPhMI molecules is known in the literature [3,25]. Taking this into account, NMR spectra were recorded for the monomers in isolation and in a mixture at various ratios. The variation in the chemical shifts in protons associated with the CPhMI molecule in the NMR spectra of CPhMI–styrene mixtures with varying compositions (A >> D) indicates the formation of a donor–acceptor [D⋯A] complex [25]. The magnitude of the chemical shift change increases with increasing molar fraction of the donor in the monomer mixture. Regardless of the initial monomer feed composition, the near-equimolar incorporation of monomer units in the copolymer provides strong evidence that the copolymerization proceeds via the formation of a donor–acceptor charge-transfer complex. The donor–acceptor complex formation can be described by the following equilibrium:

$$\left[D\right]+\left[A\right]\overset{K_{c}}{↔}\left[D\ldots A\right]$$

The equilibrium constant (Kc) of the complex formation is expressed as follows:

$$K_{c}=\frac{\left[D\ldots A\right]}{\left[D\right]\cdot \left[A\right]}$$

For the determination of the equilibrium constant, a Benesi–Hildebrand-type linearization based on NMR chemical shift changes was applied.

$$\frac{1}{A}=\frac{1}{K\varepsilon \left[A_{0}\right]}+\frac{1}{\varepsilon }$$

The corresponding data used for the calculation of the complex formation constants are presented in Table 5.

**Figure 7:**
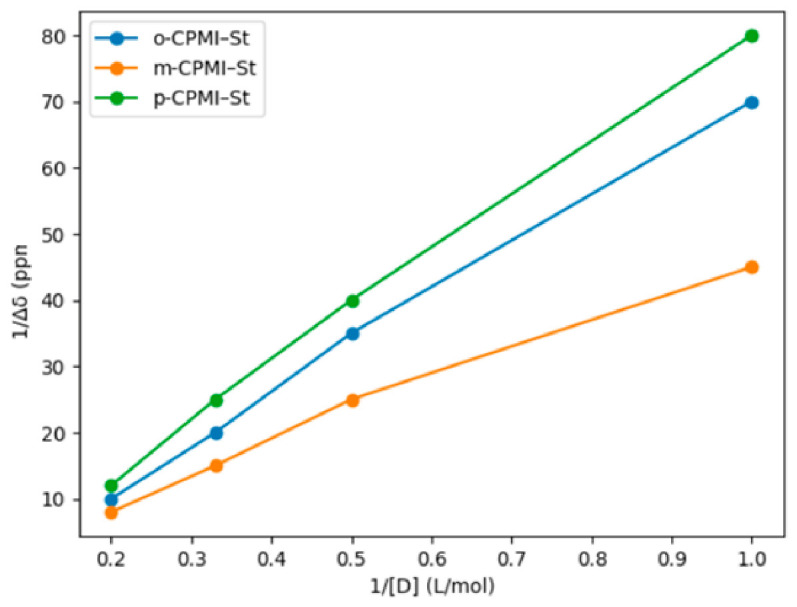
Determination of equilibrium constants for complex formation between *o*-, *m*-, and *p*-CPhMI and styrene monomer pairs using NMR spectroscopy.

According to approximate Benesi–Hildebrand estimates, the equilibrium constants for complex formation between styrene and *o*-, *m*-, and *p*-carboxyphenylmaleimide may lie in the range of 0.18–0.25 L·mol^−1^, with the tendency *p* > *m* > *o*, which can be attributed to reduced steric hindrance and more favorable intermolecular interactions in the *para*-substituted system. The equilibrium constants for complex formation between *o*-, *m*-, and *p*-carboxyphenylmaleimide and styrene monomer pairs were determined using NMR spectroscopy, as shown in Figure 7.

Thus, it can be concluded that the complex formed between the monomers has a significant influence on their relative reactivity. The dependence of the overall copolymerization rate on the composition of the monomer mixture was investigated for both monomer pairs. As shown in the figure, this dependence is non-linear and exhibits a pronounced maximum. A shift in the maximum of the curves with changes in the total monomer concentration is observed. This behavior indicates that both free monomers and their complex participate in the copolymerization process.

The association constants between *ortho*-, *meta*-, and *para*-carboxyphenylmaleimide and styrene were determined by ^1^H NMR spectroscopy using the Benesi–Hildebrand approach. Linear correlations were obtained by plotting 1/Δδ versus 1/[St], indicating the formation of 1:1 noncovalent complexes. The equilibrium constants were calculated from the ratio of the intercept to the slope of the corresponding plots. The results revealed a clear substituent-position dependence of the interaction strength.

### 3.3. Antibacterial Evaluation

The comparative characterization of the antimicrobial action of carboxyphenylmaleimide and its copolymer with styrene is shown in Table 6.

The antimicrobial activity of the synthesized CPMI–styrene copolymers was evaluated against several bacterial and fungal strains by measuring the inhibition zone diameter. The results indicate that all tested copolymers exhibit noticeable antimicrobial activity compared with the control (ethyl alcohol), which showed only minimal inhibition. Among the three copolymers, the *o*-CPMI–styrene copolymer demonstrated the highest antimicrobial effect against most tested microorganisms, with inhibition zones reaching up to 18 mm against *Candida albicans*. The *m*-CPMI–styrene copolymer showed moderate activity, while the *p*-CPMI–styrene copolymer generally displayed the lowest antimicrobial performance. *Candida albicans* was the most sensitive microorganism to all copolymers, whereas *Pseudomonas aeruginosa* and *Klebsiellapneumoniae* showed comparatively lower susceptibility. These findings suggest that the antimicrobial efficiency of CPMI–styrene copolymers is influenced by the positional isomerism of the carboxyl group in the phenyl ring, with the *ortho*-substituted copolymer providing the most effective antimicrobial activity.

### 3.4. Study of Rheological Properties of Thecopolymers

A comparative study of the viscosity of *copolymers* based on *o*-, *m*-, *p*-CPhMI–*styrene* was conducted over a range of temperatures following the methodology described in [32,33].

**Figure 8:**
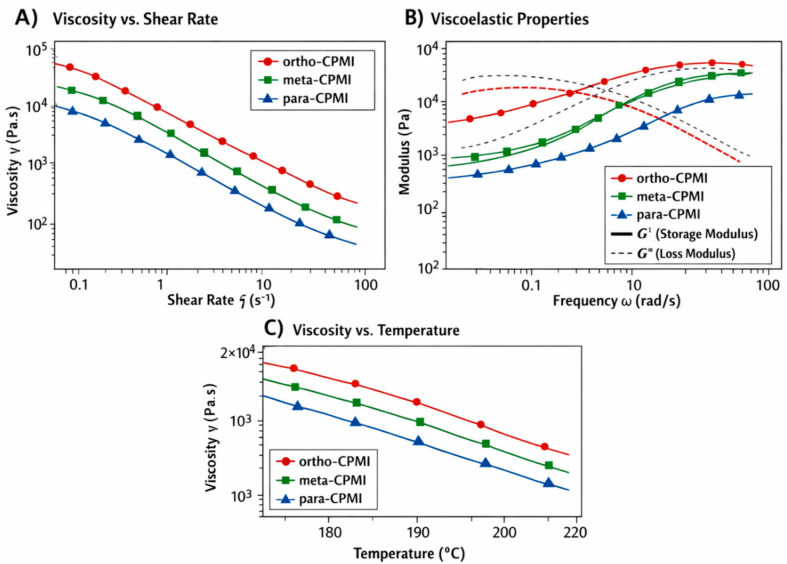
Rheological analysis of *o*-, *m*-, *p*-carboxyphenylmaleimide–styrene copolymers: viscosity as a function of shear rate (**A**); storage modulus (G′) and loss modulus (G″) as a function of angular frequency (**B**); viscosity as a function of temperature (**C**).

The viscosity–shear rate relationships of CPMI–styrene copolymers are presented in Figure 8A. All samples exhibit typical shear-thinning behavior characteristic of pseudoplastic polymer melts. Viscosity decreases with increasing shear rate due to progressive alignment of polymer chains under shear.

A clear dependence on CPMI isomer structure is observed, with viscosity following the order:

*ortho*-CPMI > *meta*-CPMI > *para*-CPMI.

The higher viscosity of the *ortho*-CPMI copolymer can be attributed to steric effects and stronger intermolecular interactions, which restrict polymer chain mobility. In contrast, *para*-CPMI copolymers exhibit lower viscosity, indicating greater chain flexibility and reduced intermolecular interactions.

The viscoelastic behavior of the copolymers was evaluated by dynamic oscillatory measurements (Figure 8B). The storage modulus (G′) and loss modulus (G″) both increase with increasing frequency, demonstrating typical viscoelastic characteristics of polymer melts.

The *ortho*-CPMI copolymer shows a more pronounced elastic response, with the storage modulus approaching or exceeding the loss modulus at lower frequencies. In comparison, the *para*-CPMI copolymer exhibits more viscous-dominated behavior over a wider frequency range. The crossover frequency increases from *ortho*- to *para*-, indicating shorter relaxation times and faster chain dynamics for the *para*-substituted copolymer.

The influence of temperature on melt viscosity is shown in Figure 8C. For all copolymers, viscosity decreases significantly with increasing temperature, reflecting enhanced chain mobility and reduced intermolecular interactions.

Despite this general trend, the *ortho*-CPMI copolymer maintains higher viscosity across the studied temperature range, suggesting stronger intermolecular interactions compared with the *meta*- and *para*-substituted systems.

### 3.5. Study of Thermal Properties of Carboxyphenylmaleimide–Styrene Copolymers

**Figure 9:**
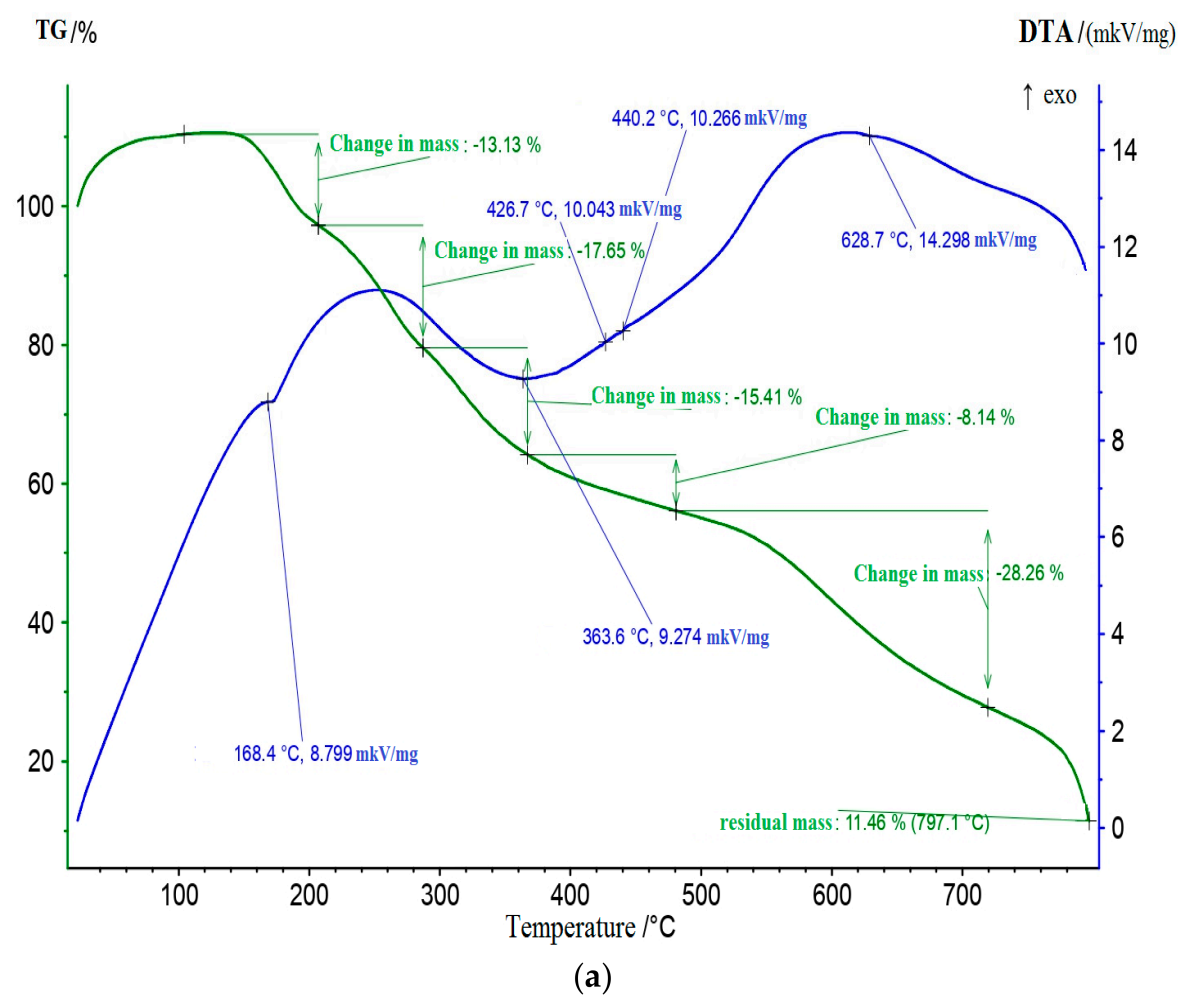

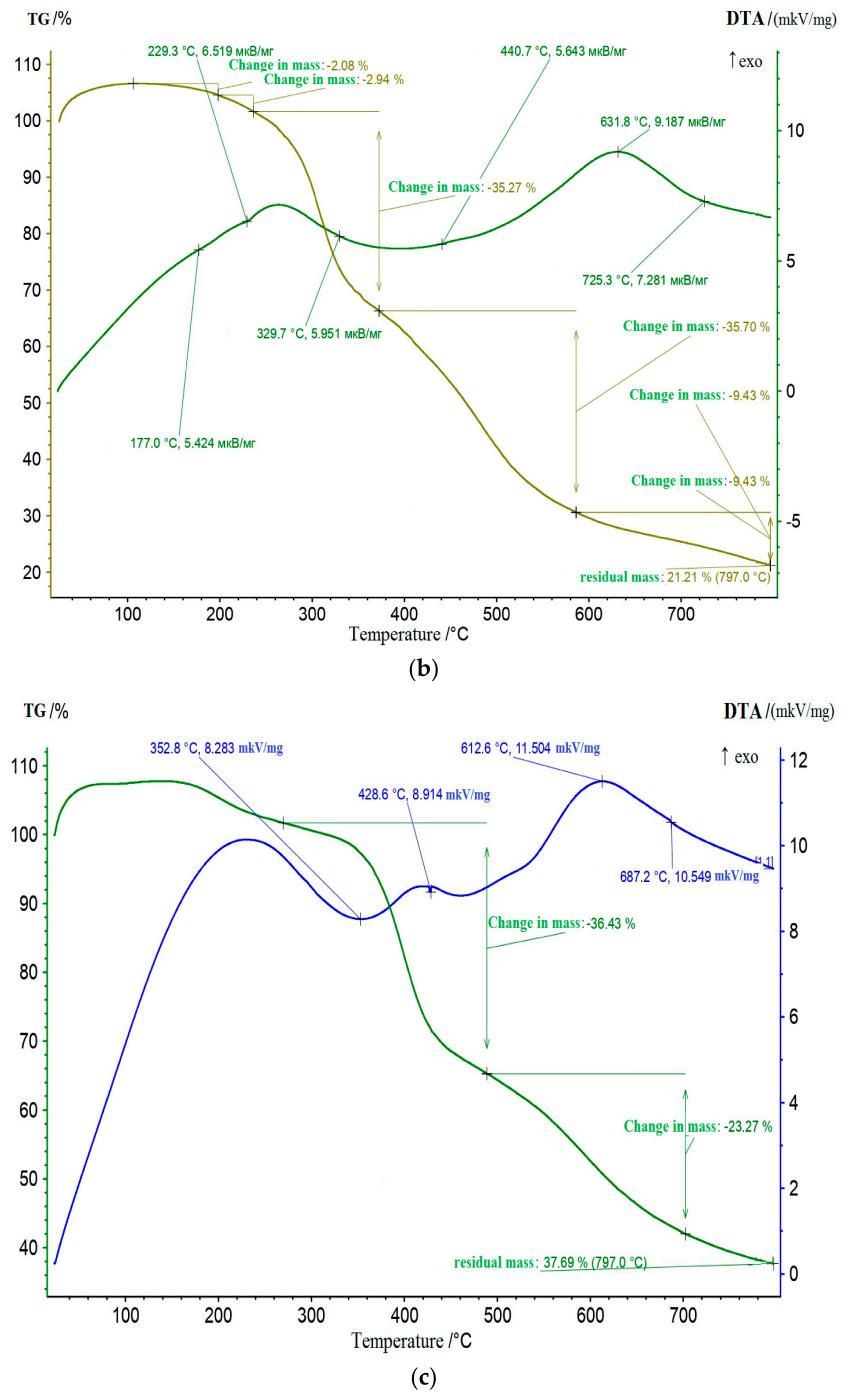

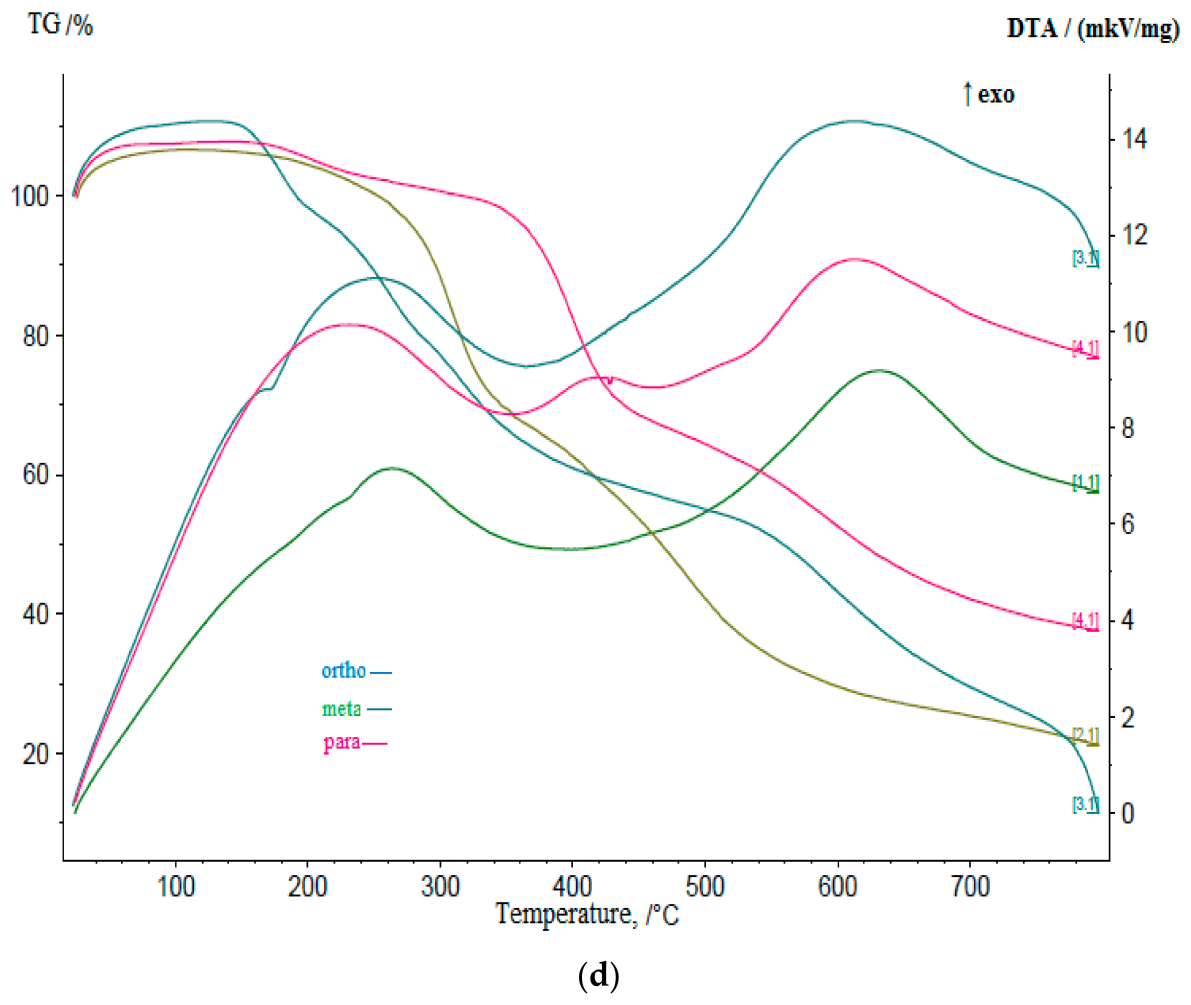
Thermogravimetric (TG) and differential thermal analysis (DTA) of *o*- (**a**), *m*- (**b**), and *p*- (**c**), comparative TG–DTG–DTA curves of *o*-, *m*-, *p*-CPhMI–St (**d**) copolymers.

The results of the differential thermal analysis (DTA) and thermogravimetric analysis (TGA) of the obtained composite materials based on *o*-, *m*-, *p*-CPhMI–St have also been determined [34,35]. The thermal behavior of the material was investigated using thermogravimetric analysis (TGA), derivative thermogravimetry (DTG), and differential thermal analysis (DTA) (Figure 9). The TGA curve (pink) shows the change in sample mass as a function of temperature, providing information on thermal stability and decomposition stages; temperature intervals with insignificant mass loss indicate thermally stable regions, while sharp decreases correspond to decomposition processes.

In this figure TG–DTA curves showing the thermal behavior of the sample as a function of temperature. The green curve represents the TG mass-loss profile, while the blue curve corresponds to the DTA heat-flow signal. Colored arrows indicate the major thermal events discussed in the text. Exo denotes the exothermic direction of the DTA signal. DTA–TG of the *ortho*-carboxyphenylmaleimide–styrene copolymer under N_2_ shows no mass loss up to 200 °C, indicating good thermal stability. An initial exothermic effect at 168 °C is attributed to structural relaxation processes. Thermal degradation begins in the range of 220–320 °C (~30.8% mass loss), which is associated with decarboxylation and side-chain cleavage. The major backbone decomposition occurs at 350–450 °C (~15.4% mass loss) accompanied by exothermic effects related to imide and styrene ring scission. Further carbonization processes take place in the range of 500–700 °C (~36.4% mass loss), resulting in a residual char of 11.5% at 797 °C. These results reflect the aromatic–imide nature of the polymer and its high thermal resistance.

DTA–TG of the *meta*-carboxyphenylmaleimide–styrene copolymer shows minor mass loss (<5%) below 250 °C, which is attributed to structural relaxation. The main thermal degradation occurs between 300 and 450 °C (~35.3% mass loss, DTA peaks at 330 and 441 °C), corresponding to maleimide decomposition, decarboxylation, and backbone scission. Further carbonization is observed in the range of 450–650 °C (~18.9% mass loss, peaks at 632 and 725 °C), leaving 21.2% residual char at 797 °C. This indicates intermediate thermal stability compared to the *ortho*- and *para*-isomers.

DTA–TG of the *para*-carboxyphenylmaleimide–styrene copolymer shows no significant mass loss up to ~300 °C, indicating excellent thermal stability. An exothermic event at 353 °C is attributed to structural rearrangement processes. Major thermal degradation occurs in the range of 350–500 °C (~36.4% mass loss, DTA peak at 429 °C), followed by further carbonization between 500 and 700 °C (~23.3% mass loss, peaks at 613 and 687 °C). A high residual mass of 37.7% at 797 °C confirms superior char formation ability due to the *para*-substituted aromatic–imide structure.

A comparative analysis of the thermogravimetric curves reveals significant differences in thermal stability among the copolymers. The *para*-substituted copolymer exhibits the highest thermal stability, as evidenced by the highest residual mass (37.7%). The *ortho*-substituted copolymer shows the lowest thermal stability with the lowest char yield (11.5%), while the *meta*-substituted copolymer demonstrates intermediate behavior with a residual mass of 21.2%.

The activation energy of thermal degradation was calculated using the double-logarithmic method described in the literature (Table 7) [40]. The onset temperature of degradation (Tn) was first determined from the TG curves. Subsequently, the mass loss (ΔG) was recorded at 10 °C intervals above Tn to evaluate the kinetic parameters of thermal decomposition.

For each temperature interval *i*, the ratio of successive mass losses was calculated as follows:

$$\frac{∆G_{i}}{∆G_{i+1}}$$

The obtained ratios were subjected to double logarithmic transformation according to the following:

$$y=ln\left[ln(\frac{∆G_{i}}{∆G_{i+1}})\right]$$

The activation energy of degradation (*Eₐ*) was calculated using the following equation:

$$E_{a}=R\cdot \tan\alpha$$

Although the para-isomer exhibited the lowest activation energy of thermal degradation, it demonstrated the highest overall thermal stability, as confirmed by its two-stage decomposition behavior and the highest residual mass (37.69%) at 800 °C. This indicates that the activation energy characterizes only the kinetic barrier of the initial degradation step, whereas the overall thermal stability is governed by the entire degradation pathway, including crosslinking, carbonization processes, and char formation.

## 4. Conclusions

A systematic investigation of the radical copolymerization of styrene with *ortho*-, *meta*-, and *para*-carboxyphenylmaleimides revealed that the copolymerization proceeds predominantly through a donor–acceptor mechanism with a pronounced tendency toward alternating sequence formation. The obtained reactivity ratios demonstrated that the position of the carboxyl substituent significantly influences monomer reactivity, with the *para*-isomer exhibiting the highest reactivity and the *ortho*-isomer the lowest, reflecting the combined effects of steric and electronic factors.

Comprehensive spectroscopic characterization (^1^H, ^13^C, and 2D NMR, FTIR, and UV–Vis spectroscopy) confirmed the successful incorporation of carboxyphenylmaleimide units into the copolymer chains, complete consumption of vinyl double bonds, and retention of the carboxyl functionality. Variations in monomer structure and composition were found to affect molecular characteristics, leading to decreases in molecular weight, intrinsic viscosity, and copolymer yield with increasing carboxyphenylmaleimide content.

The synthesized copolymers exhibited pseudoplastic flow behavior and good thermal stability, demonstrating resistance to thermal degradation up to approximately 250–300 °C. The thermal stability of the copolymers strongly depends on the substitution position. The *para*-substituted copolymer exhibits the highest thermal stability, with the highest residual mass (37.7%). The *ortho*-substituted copolymer shows the lowest thermal stability, as indicated by the lowest char yield (11.5%). The *meta*-substituted copolymer demonstrates intermediate thermal stability, with a residual mass of 21.2%. Rheological studies revealed pseudoplastic behavior for all samples, with viscosity decreasing in the order *ortho> meta > para*, indicating the influence of positional isomerism on macromolecular chain mobility and intermolecular interactions. The observed differences in rheological and thermal properties indicate that positional isomerism plays a key role in regulating intermolecular interactions, chain mobility, and degradation processes. Notably, the *para*-substituted copolymer combined enhanced copolymerization reactivity with improved char-forming ability at elevated temperatures, suggesting its suitability for the development of thermally resistant functional polymer materials.

The synthesized *o*-, *m*-, and *p*-carboxyphenylmaleimide–styrene copolymers demonstrated significant antibacterial and antifungal activity against all tested microorganisms. The strongest effect was observed against *Candida albicans*, with inhibition zones reaching up to 18 mm, while notable antibacterial activity was also observed against *Bacillus anthracoides* (up to 16 mm) and *Escherichia coli* (up to 12 mm). Among the investigated samples, the *ortho*-substituted copolymer exhibited the highest overall antimicrobial performance. The obtained results indicate a clear dependence of antimicrobial activity on the substituent position within the polymer structure. The synthesized copolymers show promising potential as functional antimicrobial polymeric materials for possible applications in coatings, additives, and related biomedical or industrial fields.

These findings establish clear structure–property relationships within the carboxyphenylmaleimide–styrene copolymer system and provide a useful basis for the molecular design of functional copolymers with tailored processing and thermal-performance characteristics.

## Figures and Tables

**Table 1:** ^1^H and ^13^C NMR chemical shifts for *o*-carboxyphenylmaleimide–styrene copolymers.

*o*-CPhMI	
δC, Type	δH (*J* in Hz)
152.8 (C-1, Ar–C–O)	3.35 (2H, br s, H-1′)
114.2 (CH, Ar–C)	2.45 (2H, t, J ≈ 7.2, H-2′)
148.6 (C-3, Ar–C–O)	2.10 (3H, s, H-3′)
130.9 (CH, Ar–C)	1.85–1.20 (m, 4H, H-4′)
129.8 (CH, Ar–C)	1.85–1.20 (m, H-5′)
128.6 (CH, Ar–C)	7.85–7.35 (4H, m, Ar–H)
165.3 (C=O)	–
140.2 (C-α, CH=)	6.52 (1H, d, J ≈ 15.6, H-α)
121.7 (C-β, CH=)	6.21 (1H, d, J ≈ 15.6, H-β)
126.3 (CH, Ar–C)	
133.7 (Cq, Ar–C)	

**Table 3:** In the copolymerization reactions of *ortho*-, *meta*-, *para*-carboxyphenylmaleimides (M_2_) and styrene (M_1_); the effect of their composition on the properties of obtained copolymers.

№	The Amount of Styrene in the Monomer Mixture, mol%	Copolymers	Amount of m_2_ in Copolymers, mol%	Yield, %	[η] dL/g	Initial Temperature of Viscous Flow, T °C
1	100	*o*-CPhMI	100	86.0	0.81	85
		*m*-CPhMI		84.0	0.78	83
		*p*-CPhMI		85.4	0.80	84
2	5.0	*o*-CPhMI	7.5	21.3	0.70	79
		*m*-CPhMI	7.2	20.5	0.66	77
		*p*-CPhMI	7.4	20.8	0.68	78
3	10.0	*o*-CPhMI	14.2	16.0	0.48	75
		*m*-CPhMI	13.8	15.2	0.43	72
		*p*-CPhMI	14.0	15.8	0.45	74
4	25.0	*o*-CPhMI	28	12.7	0.36	75
		*m*-CPhMI	27	12.0	0.30	70
		*p*-CPhMI	28	12.5	0.32	72
5	60.0	*o*-CPhMI	40	11.0	0.27	69
		*m*-CPhMI	39	10.5	0.22	66
		*p*-CPhMI	39.6	10.9	0.25	68

**Table 4:** Molecular weight characteristics of a copolymer of *p*-, *m*-, and *o*-carboxyphenylmaleimides with styrene.

Sample	T °C	Time, h	MWD			
			*M_w_*	*M_n_*	*M_w_/M_n_*	M_r_*
*o*-CPhMI-St	70	8	29,500	16,000	1.84	32,000
*m*-CPhMI-St	70	8	38,000	22,000	1.72	42,000
*p*-CPhMI-St	70	8	47,000	29,000	1.62	50,000

M_r_*—molecular mass corresponding to the peak maximum.

**Table 5:** Parameters used for the calculation of the complex formation constants (Kc) between styrene (D) and *ortho*-, *meta*-, and *para*-CPMI (A).

System	[A] mol/L	[D] mol/L	1/D l/mol	1/Δδ
*o*-CPMI–St *m*-CPMI–St *p*-CPMI–St	0.1	1.0	1.00	26.0 18.0 12.5
*o*-CPMI–St *m*-CPMI–St *p*-CPMI–St	0.1	2.0	0.50	15.0 10.5 7.5
*o*-CPMI–St *m*-CPMI–St *p*-CPMI–St	0.1	3.0	0.33	11.5 8.0 6.0
*o*-CPMI–St *m*-CPMI–St *p*-CPMI–St	0.1	4.0	0.25	9.5 6.7 5.1
*o*-CPMI–St *m*-CPMI–St *p*-CPMI–St	0.1	5.0	0.20	8.2 5.8 4.5

*o*-CPMI–St: K_c_ ≈ 0.18 L/mol, *m*-CPMI–St: K_c_ ≈ 0.20 L/mol, *p*-CPMI–St: K_c_ ≈ 0.25 L/mol.

**Table 6:** Antimicrobial activity of *o*-, *m*-, *p*-carboxyphenylmaleinimide (*o*-, *m*-, *p*-CPMI)–styrene copolymers.

Test-Culture	*o*-CPMI-Styrene Copolymer (mm)	*m*-CPMI-Styrene Copolymer (mm)	*p*-CPMI-Styrene Copolymer (mm)
*Staphylococcus aureus*	9	10	6
*Escherichia coli*	12	9	6
*Pseudomonas aeruginosa*	11	6	5
*Candida albicans*	18	16	15
*Klebsiellapneumoniae*	9	7	3
*Bacillus anthracoides*	16	13	5
Control (ethylalcohol)	3	3	3

Note: the numbers indicate the diameter of the sterile zones in mm. All experiments were repeated 3–5 times.

**Table 7:** Mass loss and activation energy during thermodestruction of *o*-, *m*-, *p*-carboxyphenylmaleimide–styrene copolymers.

Composition of the Composites (Mass %)	E_a,_ kJ·mol^−1^	Mass Loss, %				
		Temperature, °C				
		100–200	300–350	450–500	600–650	797
*o*-CPhMI + St	150	13.13	17.65	15.41	8.14	11.46
*m*-CPhMI + St	220	–	35.27	35.70	21.21	9.43
*p*-CPhMI + St	120	–	36.43	23.27	37.69	18.6

## Data Availability

The original contributions presented in this study are included in the article. Further inquiries can be directed to the corresponding author.

## Supplementary Materials

The following supporting information can be downloaded at https://www.mdpi.com/article/10.3390/polym18121507/s1, Figure S1: COSY (a), HMBC (b) NMR spectra of *o*-carboxyphenylmaleinimide–styrene copolymer; Figure S2: COSY (a), HMBC (b) NMR spectra of *m*-carboxyphenylmaleinimide–styrene copolymer.

## Abbreviations

The following abbreviations are used in this manuscript:

BP	benzoyl peroxide
CPhMI	carboxyphenylmaleimide
DTA	differential thermal analysis
MWs	molecular weights
MWD	molecular weight distribution
NMR	nuclear magnetic resonance spectroscopy
TGA	thermogravimetric analysis
IR	infrared spectroscopy
UV	ultraviolet spectroscopy
ST	styrene
*S. aureus*	*Staphylococcus aureus*
*P. aeruginosa*	*Pseudomonas aeruginosa*
*E. coli*	*Escherichia coli*
*C. albicans*	*Candida albican*
*K. pneumoniae*	*Klebsiella pneumonia*
*B. anthracoides*	*Bacillus anthracoides*

## References

[1] Yao F.J., Liu Q.Q., Zhang Z.B., Zhu X.L. (2017). RAFT Polymerization of Styrene and Maleimide in the Presence of Fluoroalcohol: Hydrogen Bonding Effects with Classical Alternating Copolymerization as Reference. Polymers.

[2] Ding J.W., Yang C.L., Zhou L.Q., Li W.J., Li J.Q., He C.X., Liu Y.F., He M., Qin S.H., Yu J. (2025). Free Radical Polymerization of Styrene and Maleimide Derivatives: Molecular Weight Control and Application as a Heat Resistance Agent. Molecules.

[3] Alikhanova A.I., Rasulzadeh N.S. (2021). Preparation and study of the properties of the copolymer of cyclic unsaturated bismaleimide with styrene. Azerbaijan Chem. J..

[4] Bag S., Ghosh S., Paul S., Khan M.E.H., De P. (2021). Styrene-Maleimide/Maleic Anhydride Alternating Copolymers: Recent Advances and Future Perspectives Macromol. Rapid Commun..

[5] Liu W., Li Q., Zhang Y., Liu T., Wang L., Li H., Hu Y. (2021). Continuous-flow RAFT copolymerization of styrene and maleic anhydride: Acceleration of reaction and effect of polymerization conditions on reaction kinetics. J. Flow Chem..

[6] Alikhanova A.I., Mamedova A.F., Ibadov E.A., Nurullayeva D.R. (2023). Preparation and study of copolymer of N, N’-(p-phenylene) bismaleimide with allyl ester of salicylic acid. Chem. Probl..

[7] Baradel N., Shishkan O., Srichan S., Lutz J.F. (2014). Synthesis of Sequence-Controlled Copolymers Using Time-Regulated Additions of N-Substituted Maleimides in Styrenic Radical Polymerizations. ACS Symp. Ser..

[8] Eken G.A., Ober C.K. (2022). Strong Polyelectrolyte Brushes via Alternating Copolymers of Styrene and Maleimides: Synthesis, Properties, and Stability. Macromolecules.

[9] Eken G.A., Kafer F., Yuan C., Andrade I., Ober C.K. (2023). Synthesis of N-Substituted Maleimides and Poly(styrene-co-N-maleimide)Copolymers and Their Potential Application as Photoresists. Macromol. Chem. Phys..

[10] Liu Q., Lv X.H., Li N., Pan X.Q., Zhu J., Zhu X.L. (2018). Copolymerization of Phenylselenide-Substituted Maleimide with Styrene and Its Oxidative Elimination Behavior. Polymers.

[11] Cozan V., Hulubei C., Airinei A., Morariu S. (2016). Maleimide copolymers containing azobenzene moieties—Synthesis and study of liquid crystalline and optical properties. RSC Adv..

[12] Cılgı G.K., Ak M. (2020). Thermal degradation kinetics and thermodynamics of maleimide-sytrene based alternating copolymer: A comparative investigation of monomer and polymer structures. J. Mol. Struct..

[13] Du W.T., Kuo S.W. (2023). Tunable thermal property of poly(styrene-alt-phenylmaleimide)-based alternating copolymers through mediated hydrogen bonding strength. Polymer.

[14] Du W.T., Orabi E.A., Mohamed M.G., Kuo S.W. (2021). Inter/intramolecular hydrogen bonding mediate miscible blend formation between near-perfect alternating Poly(styrene-alt- hydroxyphenylmaleimide) copolymers and Poly(vinyl pyrrolidone). Polymer.

[15] Hisano M., Takeda K., Takashima T., Jin Z., Shiibashi A., Matsumoto A. (2013). Sequence-controlled radical polymerization of N-substituted maleimides with 1-methylenebenzocycloalkanes and the characterization of the obtained copolymers with excellent thermal resistance and transparency. Macromolecules.

[16] Tong L., Cui X., Yang W., Deng J. (2012). Heat-resistant poly(N-(1-phenylethyl)maleimide-co-styrene) microspheres prepared by dispersion polymerization. J. Mater. Chem..

[17] Yamazaki S., Kaneko N., Kato A., Watanabe K., Aoki D., Taniguchi T., Karatsu T., Ueda Y., Motokawa R., Okura K. (2024). Synthesis of heat-resistant living polymer particles by one-step reversible addition-fragmentation chain transfer precipitation polymerization of styrene and N-phenylmaleimide. Polymer.

[18] Liu Y., He M., Yan W., Zhang D., Zhao Q., Qin S., Yu J. (2019). P(N-phenylmaleimide-alt-styrene) as a heat-resistant agent in the application of nylon 6. J. Appl. Polym. Sci..

[19] Zhao Q., Zhan Y., Liu F., He M. (2020). Synthesis of Functionalized Poly(N-(3-carboxyphenyl)maleimide-alt-styrene) and Its Heat-Resistance Mechanism. Polym. Sci. Ser. B.

[20] Garayev E.A., Guliyeva S.I., Alikhanova A.I., Mammadov B.A., Huseynguliyeva K. (2026). Obtaining and studying the properties of composite materials from *ortho*-, *meta*-, *para*-carboxyphenylmaleimide and ABS. Molecules.

[21] Alikhanova A.I., Rasulzadeh N.S., Bakhshaliyeva K.F., Muradov P.Z. (2019). Preparation and research of antibacterial polypropylene composition materials based on the cyclic bisimides. Int. J. Adv. Res. Sci. Eng..

[22] Rasulzade N.S., Alikhanova A.I., Bakhshaliyeva K.F., Muradov P.Z. (2023). Method for Obtaining Antibacterial Polymer Composite Material.

[23] Guliyeva S.I., Mammadov B.A., Garaev E.A., Alikhanova A.I., Rasulov N.S. (2025). Method for Obtaining Antibacterial Composite Materials from Carboxyphenylmaleimide and ABS.

[24] Liu Y., Jiang S., Yan W., Qin J., He M., Qin S., Yu J. (2021). Enhanced mechanical and thermal properties of polyamide 6/p(N-(4-F-phenylmaleimide)–alt-styrene) composites based on interfacial complexation inducing crystal transformation. Polymer.

[25] Guliyeva S.I., Alikhanova A.I., Rasulov N.S., Garayev E.A., Mammadov B.A. (2024). Synthesis of copolymerization of meta-carboxphenylmaleinimide with styrene and study of antimicrobial properties. Process. Petrochem. Oil Refin. (PPOR).

[26] Guliyeva S.I., Alikhanova A.I., Garayev E.A., Mammadov B.A., Rasulov N.S. (2021). Synthesis of carboxyphenymaleimides and study of their biological activity properties. Am. Sci. J..

[27] Khashami F. (2024). Fundamentals of NMR and MRI: From Quantum Principles to Medical Applications.

[28] Garbacz P. (2024). New developments in NMR. Physical Principles of Chirality in NMR.

[29] Kaye H. (2015). Encyclopedia of Infrared Spectroscopy: Volume III (Polymers, Biopolymers and Minerals Technology).

[30] El-Azazy M., Al-Saad K., El-Shafie A.S. (2023). Infrared Spectroscopy—Perspectives and Applications.

[31] Moldoveanu S., David V. (2022). Essentials in Modern HPLC Separations.

[32] Gupta R.K. (2000). Polymer and Composite Rheology.

[33] Dodero A., Williams R., Gagliardi S., Vicini S., Alloisio M., Castellano M. (2019). A micro-rheological and rheological study of biopolymers solutions: Hyaluronic acid. Carbohydr. Polym..

[34] Assad M.E.H., Khosravi A., Hashemian M. (2023). The Fundamentals of Thermal Analysis.

[35] Wagner M. (2018). Thermal Analysis in Practice, Fundamental Aspects.

[36] Murray P.R., Rosenthal K.S., Pfaller M.A. (2025). Medical Microbiology.

[37] Patravale V., Disouza J.I., Shahiwala A. (2024). Polymers for Pharmaceutical and Biomedical Applications: Fundamentals, Selection, and Preparation.

[38] Haktaniyan M., Bradley M. (2022). Polymers showing intrinsic antimicrobial activity. Chem. Soc. Rev..

[39] Olmos D., Gonzalez-Benito J. (2021). Polymeric Materials with Antibacterial Activity: A Review. Polymers.

[40] Sitnikova V.E., Ponomareva A.A., Uspenskaya M.V. (2021). Methods of Thermal Analysis: Practical.

