# Supplementary material for: Synthesis and Comparative Investigation of Ortho-, Meta-, and Para-Carboxyphenylmaleimide–Styrene Copolymers
Source: Polymers (Basel). 2026 Jun 16;18(12):1507. doi: 10.3390/polym18121507 (PMC13306373; doi:10.3390/polym18121507)
Supplement: Supplementary file 1 [file polymers-18-01507-s001.zip › polymers-4353578-supplementary.pdf]

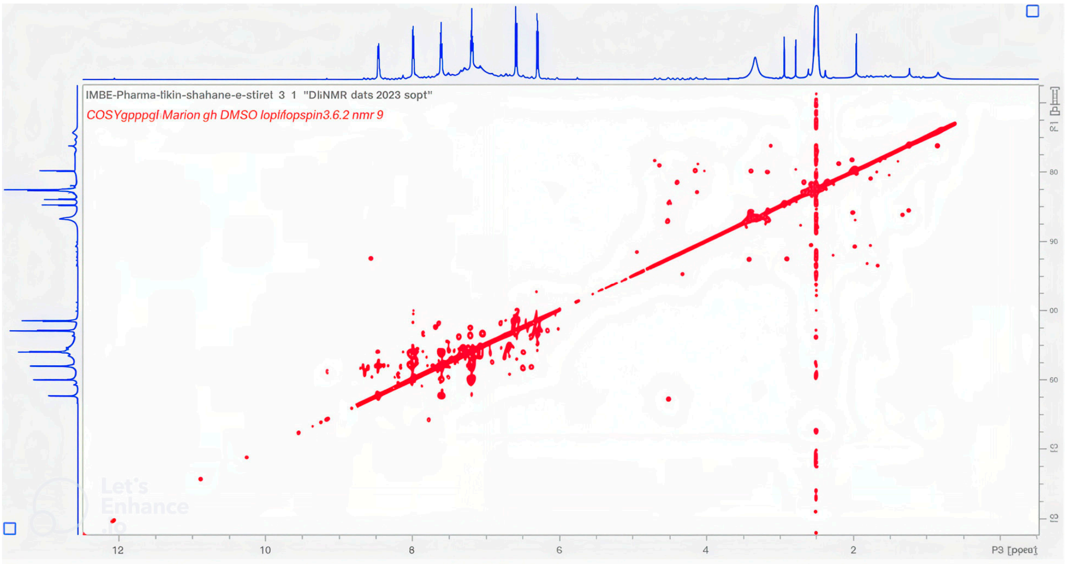

a)

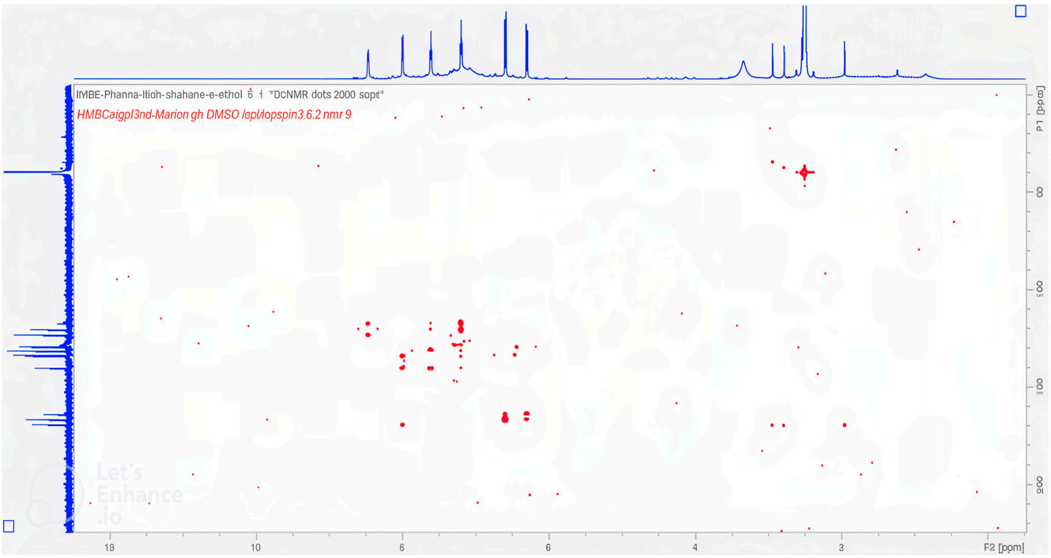

b)

Figure S1. COSY (a), HMBC (b) NMR spectra of *o*-carboxyphenylmaleinimide–styrene copolymer.

The 2D NMR spectra (COSY and HMBC) of the o-, m-CPMI–styrene copolymer are provided in the Supporting Information. COSY spectra show proton–proton correlations, while HMBC spectra display long-range proton–carbon correlations, supporting the structural characterization presented in the main manuscript.

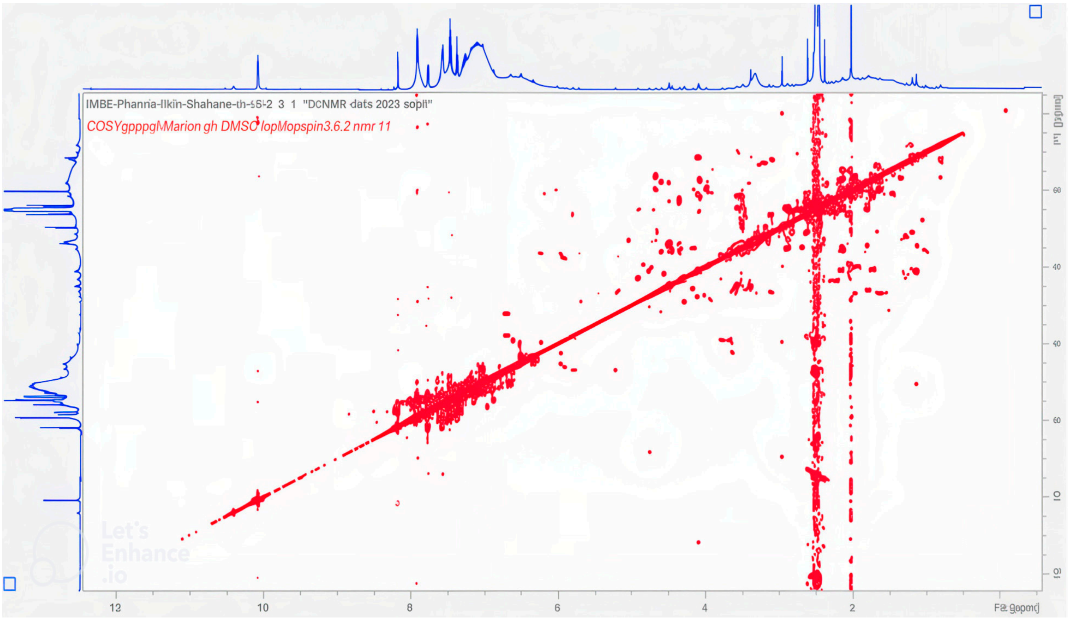

a)

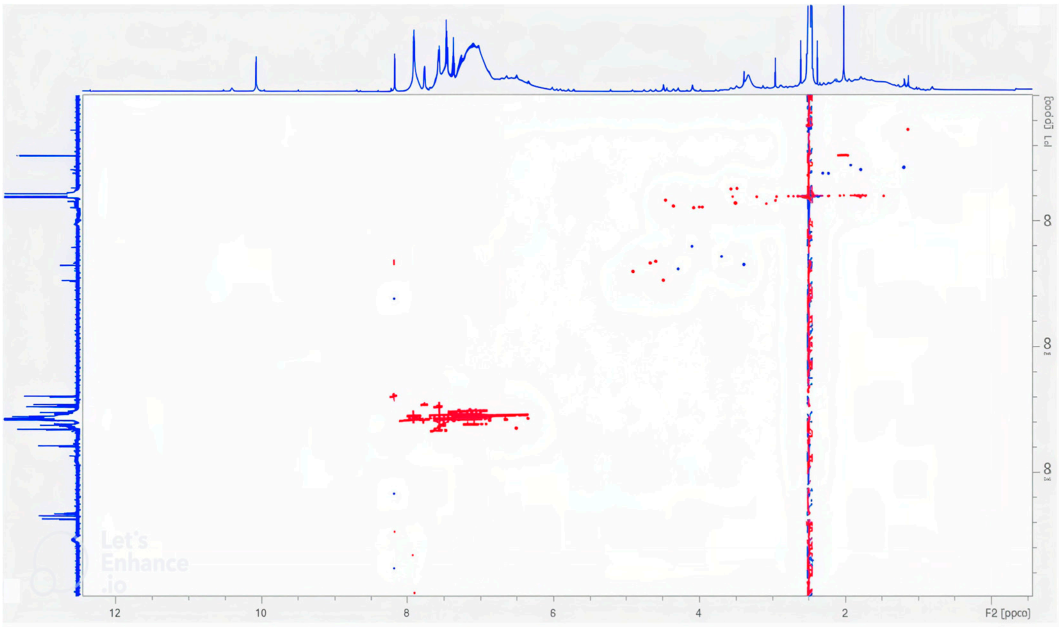

b)

Figure S2. COSY (a), HMBC (b) NMR spectra of *m*-carboxyphenylmaleinimide–styrene copolymer.

The structure of the synthesized poly(styrene-co-*meta*-carboxyphenylmaleimide) was confirmed by ²D ^1H–^1H COSY NMR. Broad aromatic signals at 6.5–8.5 ppm with cross-peaks correspond to phenyl protons of both styrene and maleimide units, reflecting polymer heterogeneity. Aliphatic correlations at 1.5–2.5 ppm arise from methine and methylene protons of the polymer backbone, confirming covalent incorporation of both monomers. The absence of vinyl proton signals indicates complete C=C bond conversion. A weak, broadened high-shift signal corresponds to carboxylic protons. Overall, COSY data

confirm successful copolymerization with preserved aromatic and carboxylic functionalities.

^{2}D HMBC NMR shows long-range ^{1}H–^{13}C correlations confirming the connectivity of styrene and meta-carboxyphenylmaleimide units. Aromatic protons (δH 7.2–8.3 ppm) correlate with quaternary carbons (δC ~130–145 ppm) and a carboxyl carbon (δC ~166–170 ppm), verifying the *meta*-carboxyphenyl group. Imide carbonyls (δC ~170–175 ppm) correlate with backbone methine/methylene protons (δH ~2.0–3.5 ppm), confirming covalent attachment. No vinyl correlations are observed, indicating complete monomer conversion. Data confirm successful copolymerization and retention of carboxylic acid functionality.
